# Transcriptome Profiling of Whole Blood Cells Identifies PLEK2 and C1QB in Human Melanoma

**DOI:** 10.1371/journal.pone.0020971

**Published:** 2011-06-15

**Authors:** Yuchun Luo, Steven Robinson, Junichi Fujita, Lisa Siconolfi, Jay Magidson, Carl K. Edwards, Karl Wassmann, Kathleen Storm, David A. Norris, Danute Bankaitis-Davis, William A. Robinson, Mayumi Fujita

**Affiliations:** 1 Department of Dermatology, University of Colorado Denver, Aurora, Colorado, United States of America; 2 Department of Medicine, University of Colorado Denver, Aurora, Colorado, United States of America; 3 Source MDx, Boulder, Colorado, United States of America; 4 Statistical Innovations, Belmont, Massachusetts, United States of America; University of Leuven, Belgium

## Abstract

**Background:**

Developing analytical methodologies to identify biomarkers in easily accessible body fluids is highly valuable for the early diagnosis and management of cancer patients. Peripheral whole blood is a “nucleic acid-rich” and “inflammatory cell-rich” information reservoir and represents systemic processes altered by the presence of cancer cells.

**Methodology/Principal Findings:**

We conducted transcriptome profiling of whole blood cells from melanoma patients. To overcome challenges associated with blood-based transcriptome analysis, we used a PAXgene™ tube and NuGEN Ovation™ globin reduction system. The combined use of these systems in microarray resulted in the identification of 78 unique genes differentially expressed in the blood of melanoma patients. Of these, 68 genes were further analyzed by quantitative reverse transcriptase PCR using blood samples from 45 newly diagnosed melanoma patients (stage I to IV) and 50 healthy control individuals. Thirty-nine genes were verified to be differentially expressed in blood samples from melanoma patients. A stepwise logit analysis selected eighteen 2-gene signatures that distinguish melanoma from healthy controls. Of these, a 2-gene signature consisting of PLEK2 and C1QB led to the best result that correctly classified 93.3% melanoma patients and 90% healthy controls. Both genes were upregulated in blood samples of melanoma patients from all stages. Further analysis using blood fractionation showed that CD45^−^ and CD45^+^ populations were responsible for the altered expression levels of PLEK2 and C1QB, respectively.

**Conclusions/Significance:**

The current study provides the first analysis of whole blood-based transcriptome biomarkers for malignant melanoma. The expression of PLEK2, the strongest gene to classify melanoma patients, in CD45^−^ subsets illustrates the importance of analyzing whole blood cells for biomarker studies. The study suggests that transcriptome profiling of blood cells could be used for both early detection of melanoma and monitoring of patients for residual disease.

## Introduction

Malignant melanoma is the most aggressive form of skin cancer, and the fifth and seventh most common cancer in men and women in the USA, respectively [1]. The American Cancer Society estimates that 68,130 new melanoma cases will be diagnosed and 8,700 people will die from melanoma in the USA in 2010 [1]. Because systemic therapies for advanced melanoma have limited efficacy, early detection and accurate staging of melanoma remains the mainstay of curative treatment of melanoma.

Genome-wide gene expression profiling has been used to better classify many cancers [2], [3] and to understand the molecular pathways involved in diverse disease processes [4], [5], [6], [7], [8], [9]. Affymetrix microarrays have been extensively used to obtain gene expression profiles from human melanoma tissues and human melanoma cells [10], [11], [12], [13], [14], [15], [16], [17], [18], [19], [20], [21]. However, obtaining fresh cancer cells and tissues from cancer patients for laboratory analysis is sometimes challenging for primary tumor whose lesion is usually small and whose entire lesion needs to be formalin-fixed for accurate diagnosis and staging. Therefore, developing analytical methodologies to detect and identify biomarkers in easily accessible body fluids such as peripheral blood would be highly valuable for the early diagnosis and management of cancer patients [22].

Peripheral whole blood is a “nucleic acid-rich” and “inflammatory cell-rich” information reservoir. Anti-tumor responses are frequently observed in the blood of cancer patients whereas immune systems often facilitate tumor progression by sculpting the immunogenic phenotype of tumors (immunoediting) and by secreting cytokines and inflammatory elements, proteases and other extracellular matrix modulators [23], [24], [25], [26], [27]. In addition to immunocytes, peripheral blood of cancer patients contains circulating tumors cells, endothelial cells, and bone-marrow-derived cells, all of which can be used as resources for molecular biomarkers. We hypothesized that peripheral whole blood represents systemic processes altered by the presence of cancer cells in the tumor microenvironment and/or in the circulation, and that analytical methodologies to detect phenotypic changes of these cells in the blood will provide relevant biomarkers in cancer patients.

Blood-based gene expression biomarkers have been investigated in various non-cancerous disease conditions such as autoimmune, infectious, and neurological diseases [7], [8], [28], [29], [30], [31], [32]. Blood transcript profiling has also been studied in human cancers using peripheral blood mononuclear cells [33], [34], [35], [36], [37]. Furthermore, circulating T cells [38] and tumor cells [39], [40] isolated from blood have been investigated by gene expression analysis in melanoma patients and malignant glioma patients [41]. While these studies have provided valuable information on blood cell-derived gene expression from cancer patients, the information sometimes show variance due to the analysis of a subpopulation of blood. Pre-analytical steps such as storing, handling and isolation of blood may induce changes in gene expression. Furthermore, multiple steps before analysis in laboratories may hinder future application of biomarker studies for multi-center clinical trials. Collecting whole blood cells directly into a PAXgene™ RNA stabilization tube instantly stabilizes RNA, reduces process-related artifacts and has been used to profile blood transcriptomes from colon and breast cancer patients [42], [43], [44]. However, the abundance of globin mRNA in whole blood RNA preparations interferes with hybridization efficiency of non-globulin transcripts when performing microarray analysis of whole blood cells, resulting in reduced present calls and augmented variability in microarray analysis [45]. Several methods have been developed to reduce the impact of globin RNA including an Ambion GLOBINclear kit, Affymetrix peptide nucleic acid methodology and NuGEN Ovation™ technology [46], [47], [48].

In the present study, we used the PAXgene™ RNA stabilization tube to instantly stabilize RNA from whole blood. We also used biotinylated probes from the NuGEN Ovation™ Whole Blood Solution to amplify non-globin RNA without the impact of globin RNA for microarray analysis. The combined use of these systems resulted in the identification of unique genes differentially expressed in the blood of melanoma patients. Genes selected from microarray analysis were verified by high-throughput quantitative reverse transcriptase PCR (qRT-PCR) analysis and 39 genes were confirmed to be expressed in melanoma blood. A stepwise logit analysis selected eighteen 2-gene signatures that distinguish melanoma from healthy controls. Of these, a 2-gene signature consisting of PLEK2 and C1QB led to the best result that correctly classified 93.3% melanoma patients and 90% healthy controls. Both genes were upregulated even in blood samples from stage I and II melanoma patients. Further analysis using blood fractionation showed that CD45^−^ and CD45^+^ populations were responsible for the altered expression levels of PLEK2 and C1QB, respectively, indicating the importance of analyzing whole blood cells for blood-based biomarker studies of cancer.

## Methods

### Ethics statement

All blood samples were collected at the University of Colorado Hospital. The study protocol was approved by the University of Colorado Hospital Institutional Review Board (#05-0309). Written informed consents were obtained from all participants who agreed to serve as blood donors.

### Patient samples

One hundred fifty-one blood samples were collected for this study (69 from melanoma patients and 82 from healthy controls). Of these 151 samples, 10 samples were used in microarray analysis, 95 samples were used in high-throughput qRT-PCR analysis, 6 samples were used to identify cell subsets in fractionated blood samples and 40 samples were used to compare genes in fractionated blood samples. Melanoma patients were primarily of Caucasian descent, and blood samples from age- and sex-matched control were obtained from healthy Caucasians. For the microarray analysis, whole blood samples were obtained from 4 newly diagnosed melanoma patients (stage IV, without treatment) and 6 healthy individuals. For the high-throughput qRT-PCR analysis, whole blood samples were collected from 45 newly diagnosed melanoma patients (stage I to IV) and 50 healthy individuals. Of the 45 melanoma patients, 5 were diagnosed with stage I melanoma, 8 were diagnosed with stage II melanoma, 11 were diagnosed with stage III melanoma and 21 were diagnosed with stage IV melanoma. For the analysis of fractionated samples to identify cell subsets responsible for the expression of genes, heparin-treated blood samples were obtained from 6 healthy control subjects. For the analysis of fractionated samples to compare genes, heparin-treated blood samples were obtained from 20 newly diagnosed melanoma patients and 20 healthy control subjects. Of the 20 melanoma patients, 8 were diagnosed with stage I melanoma, 3 were diagnosed with stage II melanoma, 3 were diagnosed with stage III melanoma and 6 were diagnosed with stage IV melanoma. Blood samples were collected with the written informed consent of patients under IRB approved protocols and adhering to HIPAA regulations.

### Whole blood collection and RNA isolation

Whole blood samples were collected into a PAXgene™ RNA stabilization tube (PreAnalytiX, Hombrechtikon, CH) (2.5 ml). The tube contains a nucleic acid preservative and provides a means for collection, stabilization and transportation of a whole blood cellular RNA specimen in a closed evacuated system. RNA was extracted from the samples using a PAXgene™ Blood RNA Kit (PreAnalytiX) in accordance with the manufacturer's protocol. Quality of the RNA was verified on an Agilent® 2100 Bioanalyzer (Agilent Technologies, Palo Alto, CA) and the quantity of RNA was determined by NanoDrop® ND-1000 spectrophotometer (Thermo Scientific, Wilmington, DE).

### Blood cell fractionation and RNA isolation

Fresh blood samples were collected using Vacutainer® Heparin Tubes (BD, Franklin Lakes, NJ) and kept on ice, followed by labeling with MACS human CD45^+^ microbeads (Miltenyi Biotec, Auburn, CA). Briefly, whole blood (3 ml) was added with 150 µl microbeads and incubated for 15 minutes at 4°C. Labeled cells were washed and resuspended in autoMACS Running Buffer, followed by separation with autoMAC™ separator (Miltenyi Biotec). Program “posseld2” and “depletes” were used to isolate CD45-positive (CD45^+^) and CD45-negative (CD45^−^) cells, respectively. RNA was immediately extracted from fractionated cells by Qiagen RNeasy mini kit (Qiagen, Valencia, CA).

### Microarray hybridization

Total RNA (50 ng) from whole blood samples was amplified using Ovation™ Biotin RNA Amplification and Labeling System V1 (NuGEN Technologies, San Carlos, CA) according to the manufacturers' instructions. Labeled cDNA was hybridized to Affymetrix Human Genome U133 Plus 2.0 GeneChip oligonuceotide arrays (54,000 probe sets, >47,000 transcripts) (Affymetrix, Santa Clara, CA). Hybridization signals were adjusted using Affymetrix Genechip® Operating Software (GCOS) (version 1.1.1). GeneSpringGX version 10.0 (Agilent Technologies, Santa Clara, CA) was used to analyze microarray data for hierarchical clustering and significant pathways.

### Quantitative RT-PCR (qRT-PCR) analysis

RNA from whole blood samples was analyzed for the expression of 68 genes by high-throughput qRT-PCR whereas RNA from fractionated samples was analyzed for the expression of 2 genes by standard qRT-PCR.

High-throughput qRT-PCR was performed at Source MDx (SMDx, Boulder, CO). Briefly, first strand cDNA was synthesized from RNA by reverse transcription following priming with random hexamers. Primer/probe reagents selected from the microarray data were custom designed with the aid of Applied Biosystems Primer Express® Software (Carlsbad, CA) following SMDx proprietary design specifications. Precision profile melanoma microarray plates were manufactured using a high-throughput Biomek® FX Laboratory Automation Workstation (Beckman Coulter, Brea, CA) and the primer/probe sets of each target gene of interest resided in triplicate wells. Rigorous quality control testing ensured that amplification specificity and efficiency were within defined limits. To carry out the assays, cDNA from samples was added to the Precision Profile plates and high-throughput qRT-PCR was performed. The intensity of released fluors was measured as a function of time and compared with parallel analysis to determine the background level. Each PCR reaction contained primer/probe sets for the target gene and 18S RNA, used as an internal control. The difference between the fluorescence threshold cycle (C_T_) for the target and the internal endogenous control (18S) was presented as a ΔC_T_ value.

Standard qRT-PCR was performed at the University of Colorado Denver. Fractionated cells were analyzed for the expression of PLEK2 and C1QB by standard qRT-PCR. Three pairs of primers were used: PLEK2-F: 5′-GTGCTCAAGGAGGGCTTC-3′; PLEK2-R: 5′-GCTTGTAGTACACCAGCGTGTT-3′; C1QB-F: 5′-AAGGTGCCCGGTCTCTACTA-3′; C1QB-R: 5′-ACCTGGAAGGTGTTGTAGGC-3′; GAPDH-F: 5′-TGCACCACCAACTGCTTAGC-3′; GAPDH-R: 5′-GGCATGGACTGTGGTCATGAG-3′. PCR amplification and quantitation was performed using ABI SYBR Green Master Mix (Applied Biosystems, Foster City, CA) and Stratagene MX3000P™ (Cedar Creek, Texas). GAPDH was used as an internal control for normalization.

### Statistical analysis

One-way ANOVA and logistic regression were used to evaluate expression differences of high-throughput qRT-PCR between melanoma patients and healthy controls. For one-way ANOVA, statistical measures were determined using Enterprise Guide version 2.05.89 (SAS Institute, Inc., Cary, NC). The Anderson-Darling test and the Shapiro-Wilk test were used to determine whether the gene expression data fit a normal distribution. Student's t-tests were performed to determine *p*-values and genes were ranked in the order of significance using a one-WAY ANOVA approach. Signature models distinguishing melanoma patients from healthy control individuals were generated using an automated search procedure in the program GOLDMineR® (Graphical Ordinal Logit Displays based on Monotonic Regression) that implements a stepwise logit analysis for selecting the predictor variables. All predictor genes were first ranked according to their unique *p*-values. The most significant gene that distinguished melanoma from healthy controls was first entered into the logit analysis. Additional genes were added one step at a time in a fast, stepwise inclusion algorithm process generated models of prediction based on target genes assayed. Multiple models were generated and evaluated based on goodness of prediction (R^2^, coefficient of determination). The cutoff of R^2^ value was 0.6.

To evaluate expression levels of fractionated blood samples, Student's t*-*test was used. *p*-values of <0.05 were considered statistically significant.

## Results

### Identification of differentially expressed genes using microarray

**Figure 1:**
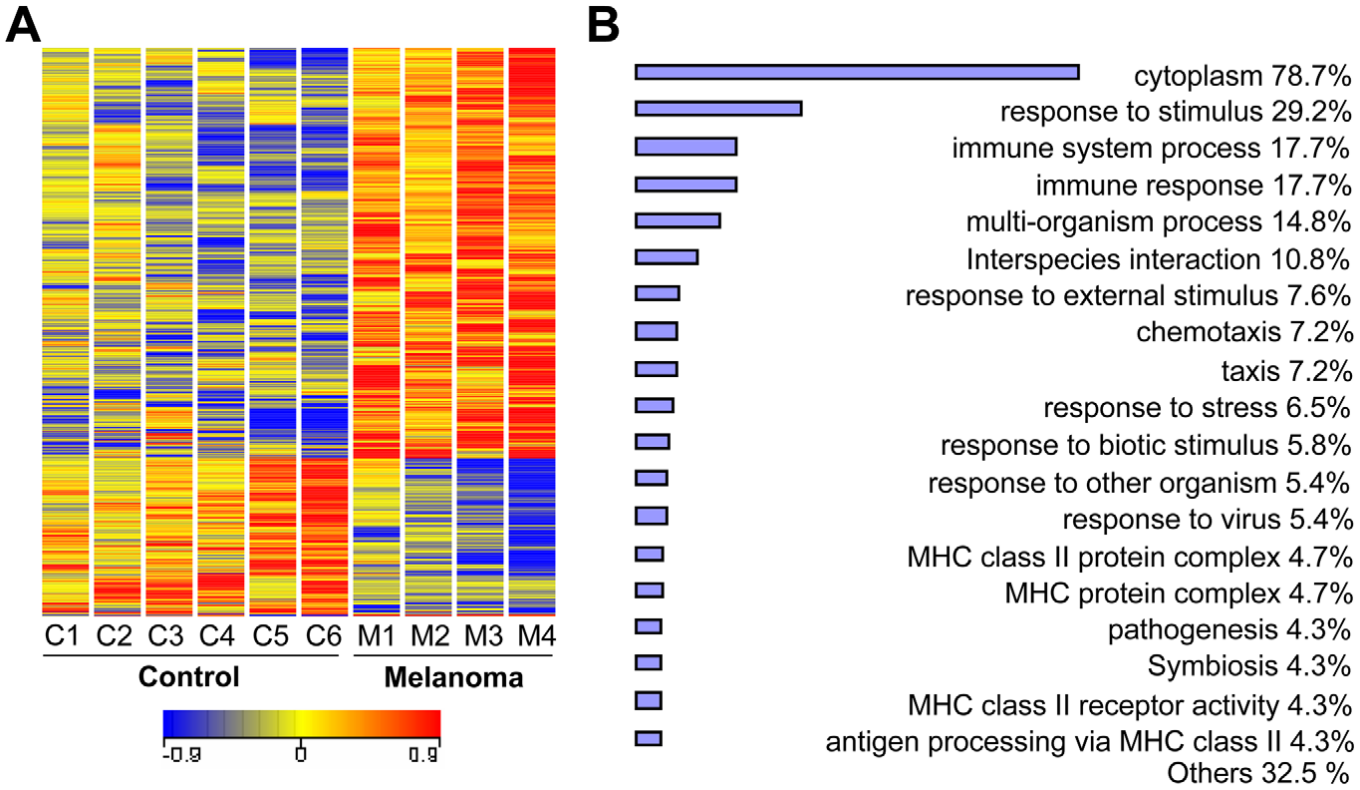
Microarray analysis of differentially expressed genes in melanoma. **A.** Hierarchical clustering of differentially expressed genes. Whole blood samples were obtained from 4 newly diagnosed melanoma patients (stage IV, with no treatment, M1–M4) and 6 healthy control individuals (C1–C6). Clustering of 1187 transcripts in blood from melanoma patients and healthy control individuals (*p*<0.05). Colored spots indicate significant (*p*<0.05) upregulation (red) or downregulation (blue) of transcripts. Sample tree originated from the clustering of values with Euclidean distance analyzed by GeneSpringGX 10.0. **B.** Gene ontology analysis of differentiated expressed genes (*p*<0.05) between melanoma patients and healthy control individuals. Bar chart was drawn using gene ontology (*p*<0.01) tool from GeneSpringGX 10.0.

Whole blood samples from 4 newly diagnosed melanoma patients (stage IV, without treatment) and 6 healthy individuals were collected into PAXgene™ RNA stabilization tubes. Isolated RNA from the samples was of high quality with intact 18 s and 28 s ribosomal RNA and A_260/280_ of 2.1–2.2. Using Affymetrix GeneChip U133A, we analyzed global gene expression changes in blood samples from stage IV melanoma patients and healthy individuals. A comparative analysis identified 997 significantly altered transcripts (*p*<0.05, expression ratio >1.8 or 0.6). Hierarchical cluster analysis of these genes showed clear differences between melanoma patients and healthy individuals (Fig. 1A). Differentially expressed genes were then subjected to gene ontology analysis to identify biological processes (Fig. 1B). The analysis revealed that multiple processes were significantly altered in blood of melanoma patients compared with that from healthy individuals (*p*<0.01). Many genes were associated with cytoplasmic processes (78.7% of genes) and response to stimulus (29.2%). Particularly, several immunological processes were identified to be differentially expressed in blood samples from melanoma patients including immune system process (17.7%), immune response (17.7%), chemotaxis (7.2%), MHC class II protein complex (4.7%), MHC protein complex (4.7%), MHC class II receptor activity (4.3%) and antigen processing via MHC class II (4.3%). Differentially expressed genes (997 genes) were also subjected to significant pathway analysis, which identified three significant (*p*<0.01) pathways: IL-4, Notch and Wnt (Fig. S1).

### Selection of candidate genes by microarray analysis

We selected 78 candidate genes based on the criteria using fold changes, expression levels and comparison replicates. Transcripts shown in Fig. S2A (63 genes) were present above threshold level (“present” call, >10 ppm), had 1.8-fold or greater significant differences between healthy control individuals and melanoma patients, and had a high score by comparison replicates. Transcripts shown in Fig. S2B had 5.5-fold or greater differences between healthy individuals and melanoma patients. Most of the candidate genes did not express high levels of transcripts, and the top 15 genes were selected based on comparison replicates. Of the 78 selected genes, 46 were upregulated and 32 were downregulated (Table S1). The gene list was submitted to SMDx for further validation by high-throughput qRT-PCR.

### Verification of candidate genes using high-throughput qRT-PCR analysis

High-throughput qRT-PCR was performed using whole blood samples obtained from 45 newly diagnosed melanoma patients (stage I to IV) and 50 healthy individuals (see Methods). Of the 78 selected genes selected from microarray analysis, primers/probes of 68 genes were custom designed at SMDx. Precision profile melanoma plates were manufactured using the 68 genes and evaluated for their ability to discriminate between healthy controls and melanomas. The order of significance was ranked using a one-way ANOVA approach. Thirty-nine genes (27 upregulated genes and 12 downregulated genes) were confirmed to be differentially expressed in melanoma blood compared with healthy control blood (Table 1). The analysis yielded comparable *p*-values and comparable rankings of the genes in terms of statistical significance.

**Table 1:** List of genes identified by microarray analysis and validated by high-throughput qRT-PCR.

Gene Symbol	Gene Title	*p*-value(microarray)	*p*-value(qRT-PCR)	R2(qRT-PCR)
**Upregulated Genes**				
BLVRB	Biliverdin reductase B (flavin reductase (NADPH))	0.0322553	0.000087	0.14
BPGM	2,3-bisphosphoglycerate mutase	0.0351863	0.000029	0.1611
C1QB	Complement component 1, q subcomponent, B chain	0.00673087	5.9E-08	0.2345
C20orf108	Chromosome 20 open reading frame 108	0.0001927	0.024	0.0504
CARD12	Caspase recruitment domain family, member 12	0.00647157	0.04	0.044
CHPT1	Choline phosphotransferase 1	0.0414433	0.0034	0.0863
F5	Coagulation factor V (proaccelerin, labile factor)	0.0349511	0.011	0.064
GLRX5	Glutaredoxin 5 homolog (S. cerevisiae)	0.00303576	0.00072	0.1095
GYPA	Glycophorin A (MNS blood group)	0.00176843	0.018	0.0562
GYPB	Glycophorin B (MNS blood group)	0.0104615	0.018	0.0556
IGF2BP2	Insulin-like growth factor 2 mRNA binding protein 2	0.0166351	6.5E-06	0.1788
IL1R2	Interleukin 1 receptor, type II	0.00972327	0.0026	0.0908
IRAK3	Interleukin-1 receptor-associated kinase 3	0.00052347	0.016	0.0599
LGALS3	Lectin, galactoside-binding, soluble, 3 (galectin 3)	0.0045688	0.00047	0.1164
NEDD4L	Neural precursor cell expressed, developmentally down-regulated 4-like	0.0157874	2.5E-09	0.2787
NEDD9	Neural precursor cell expressed, developmentally down-regulated 9	0.0386422	0.009	0.0669
NUDT4	Nudix (nucleoside diphosphate linked moiety X)-type motif 4	0.00417398	0.000015	0.1684
PBX1	Pre-B-cell leukemia transcription factor 1	0.00458616	0.0052	0.0774
PLAUR	Plasminogen activator, urokinase receptor	0.0351704	0.043	0.0419
PLEK2	Pleckstrin 2	0.00079183	4.2E-22	0.4979
PLXDC2	Plexin domain containing 2	0.00322457	5E-09	0.2637
SIAH2	Seven in absentia homolog 2 (Drosophila)	0.0006551	0.000003	0.1927
SLC4A1	Solute carrier family 4, anion exchanger, member 1 (erythrocyte membrane protein band 3, Diego blood group)	0.0107523	0.000029	0.1548
TMOD1	Tropomodulin 1	0.00191409	0.0016	0.0959
TNS1	Tensin 1	0.0276222	0.013	0.0612
TSPAN5	Tetraspanin 5	0.0375013	0.0013	0.0998
XK	X-linked Kx blood group (McLeod syndrome)	0.00753119	9.8E-07	0.2066
**Downregulated Genes**				
CNKSR2	Connector enhancer of kinase suppressor of Ras 2	0.007204	0.000021	0.1681
EDIL3	EGF-like repeats and discoidin I-like domains 3	0.018857	0.00062	0.1113
INPP4B	Inositol polyphosphate-4-phosphatase, type II, 105 kDa	0.000733	0.0042	0.084
KCNK2	Potassium channel, subfamily K, member 2	0.024077	3.8E-08	0.2452
LARGE	Like-glycosyltransferase	0.000698	3.5E-06	0.1885
NBEA	Neurobeachin	0.005041	0.000017	0.1677
NUCKS1	Nuclear casein kinase and cyclin-dependent kinase substrate 1	0.038581	0.007	0.0732
PTPRK	Protein tyrosine phosphatase, receptor type, K	0.044927	0.000083	0.1473
SCAND2	SCAN domain containing 2	0.011046	0.000026	0.1623
ST6GALNAC5	ST6 (alpha-N-acetyl-neuraminyl-2,3-beta-galactosyl-1,3)-N-acetylgalactosaminide alpha-2,6-sialyltransferase 5	0.005612	0.017	0.0592
TLK2	Tousled-like kinase 2	0.012065	0.034	0.0462
ZBTB10	Zinc finger and BTB domain containing 10	0.038739	0.00088	0.1087

### Generation of signature models to distinguish melanoma patients from healthy individuals

**Figure 2:**
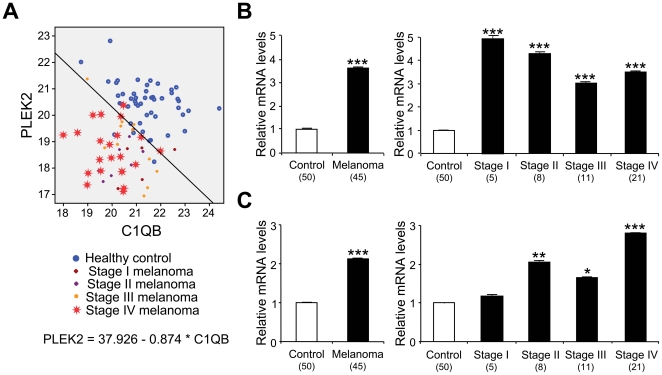
Expression of PLEK2 and C1QB in whole blood of melanoma patients and healthy control individuals by high-throughput qRT-PCR. **A.** Two-gene model of PLEK2 and C1QB. The level of gene expression was evaluated using healthy control (*n* = 50), stage I melanoma (*n* = 5), stage II melanoma (*n* = 8), stage III melanoma (*n* = 11) and stage IV melanoma (*n* = 21). PLEK2 for each subject (y-axis) is plotted against C1QB (x-axis), and the best fit line demarcating healthy controls from melanoma patients is drawn. **B.** High-throughput qRT-PCR of PLEK2. The gene expression was evaluated using healthy control (*n* = 50), stage I melanoma (*n* = 5), stage II melanoma (*n* = 8), stage III melanoma (*n* = 11) and stage IV melanoma (*n* = 21), and normalized to the level from healthy controls. **C.** High-throughput qRT-PCR of C1QB. The gene expression was evaluated using healthy control (*n* = 50), stage I melanoma (*n* = 5), stage II melanoma (*n* = 8), stage III melanoma (*n* = 11) and stage IV melanoma (*n* = 21), and normalized to the level from healthy controls. The data are shown as means ± S.E. (Sample numbers analyzed are included in parentheses). *, *p*<0.05; **, *p*<0.01; ***, *p*<0.001 compared with healthy controls.

The genes confirmed by high-throughput qRT-PCR were individually subjected to a stepwise logit analysis to select predictor genes that distinguish melanoma from controls at the Statistical Innovations. The analysis was performed using individual genes demonstrating the best power to discriminate controls from melanoma patients, as starting points. Additional genes were added in a stepwise fashion until specificity/sensitivity reached the 75% criteria and R^2^ value hit 0.6 cutoff (Table 2). Because the goal of the present study was to generate signature models that were simple enough to be translated into clinical practice, further genes were not added once the criteria and cutoff were met. Of significant importance is PLEK2 having the lowest *p*-value and a high R^2^ (0.56) that was already close to the cutoff value 0.6. Indeed, PLEK2 as a single gene model, allowed 42/50 (84%) controls and 38/45 (86.7%) melanoma patients to be classified accurately (Table 3). Therefore, PLEK2 was chosen to be a starting point (Table 2). The addition of the other gene resulted in eighteen two-gene models that met the criteria and cutoff to correctly discriminate melanoma patients and healthy control individuals (Table 3). Among the above two-gene models, the addition of C1QB led to the best result that correctly classified 93.3% (42/45) newly diagnosed melanoma patients and 90% (45/50) healthy control individuals (Table 2, Table 3, Fig. 2A). Since these genes were initially selected using microarray analysis of blood samples from metastatic melanoma (stage IV) patients, we investigated if the gene expression changes were detected in samples from early stages of melanoma. As shown in Fig. 2B and 2C, both PLEK2 and C1QB were significantly upregulated even in the blood of stage I/II patients, indicating their potential as a blood-based biomarker in early diagnosis.

**Table 2:** Stepwise logistic regression (STEP) analysis signature models of melanoma.

Gene	STEP	*p*-value	R square	Gene	STEP	*p*-value	R square
PLEK2	1	1.3E-15	0.5542	PLEK2	1	1.3E-15	0.5542
NEDD4L	1	1.6E-08		C1QB	2	2.5E-07	0.7314
PLXDC2	1	8.9E-08		PLXDC2	2	1.1E-05	
C1QB	1	3.6E-07		TMOD1	2	1.5E-05	
KCNK2	1	4.1E-07		TSPAN5	2	0.0001	
XK	1	2.3E-06		GLRX5	2	0.00012	
LARGE	1	5.6E-06		C20ORF108	2	0.00017	
SIAH2	1	5.9E-06		GYPA	2	0.00029	
IGF2BP2	1	1.3E-05		GYPB	2	0.0014	
CNKSR2	1	1.4E-05		BLVRB	2	0.0017	
NBEA	1	2.6E-05		IL1R2	2	0.0031	
NUDT4	1	2.6E-05		PBX1	2	0.0062	
SCN3A	1	3.4E-05		LARGE	2	0.0075	
BPGM	1	3.8E-05		PLAUR	2	0.012	
PTPRK	1	5.1E-05		SLC4A1	2	0.013	
SLC4A1	1	6.0E-05		KCNK2	2	0.018	
BLVRB	1	0.00015		SCN3A	2	0.02	
LGALS3	1	0.00062		PTPRK	2	0.022	
EDIL3	1	0.00082		CARD12	2	0.025	
ZBTB10	1	0.00082		TLK2	2	0.031	
GLRX5	1	0.00091		SLA	2	0.037	
INPP4B	1	0.0013		RBMS1	2	0.038	
TSPAN5	1	0.0015		CNKSR2	2	0.039	
IL1R2	1	0.0018		CXCL16	2	0.045	
TMOD1	1	0.0019		IRAK3	2	0.057	
CHPT1	1	0.0034		RAB2B	2	0.084	
PBX1	1	0.0056		NOTCH2	2	0.11	
NUCKS1	1	0.007		F5	2	0.11	
NEDD9	1	0.01		IGF2BP2	2	0.14	
F5	1	0.012		CELSR1	2	0.14	
TNS1	1	0.014		NUDT4	2	0.17	
IRAK3	1	0.014		NEDD4L	2	0.17	
ST6GALNAC5	1	0.016		BPGM	2	0.18	
GYPA	1	0.019		LGALS3	2	0.18	
GYPB	1	0.02		EDIL3	2	0.2	
C20ORF108	1	0.026		PLEKHQ1	2	0.22	
TLK2	1	0.035		ACOX1	2	0.24	
CARD12	1	0.038		HECTD2	2	0.24	
PLAUR	1	0.044		IL13RA1	2	0.25	

**Table 3:** Two-gene models identified by STEP analysis.

Model	R2	Detection rate of control individuals (%)	Detection rate of melanoma patients (%)
PLEK2-C1QB model	0.728	90.0	93.3
PLEK2-TMOD1 model	0.701	88.0	93.3
PLEK2-PLXDC2 model	0.701	88.0	93.3
PLEK2-TSPAN5 model	0.670	90.0	93.3
PLEK2-C20ORF108 model	0.662	92.0	86.7
PLEK2-GLRX5 model	0.661	90.0	88.9
PLEK2-GYPA model	0.650	88.0	91.1
PLEK2-SLC4A1 model	0.623	88.0	91.1
PLEK2-IL-1R2 model	0.621	88.0	88.9
PLEK2-BLVRB model	0.619	90.0	91.1
PLEK2-GYPB model	0.619	86.0	84.4
PLEK2-PBX1 model	0.611	88.0	86.7
PLEK2-PTPRK model	0.610	90.0	86.7
PLEK2-PLAUR model	0.603	84.0	84.4
PLEK2-CXCL16 model	0.603	88.0	88.9
PLEK2-CNKSR2 model	0.602	88.0	86.7
PLEK2-SCN3A model	0.602	86.0	86.7
PLEK2-LARGE model	0.602	88.0	84.4
PLEK2 (1-gene model)	0.560	84.0	86.7

### Identification of cell subsets responsible for the expression of PLEK2 and C1QB by blood cell fractionation

**Figure 3:**
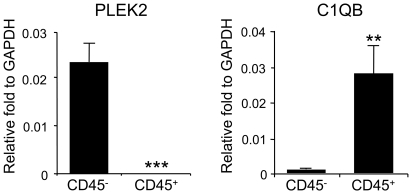
Expression of PLEK2 and C1QB in fractionated blood. Freshly collected blood cells were fractionated into CD45^+^ and CD45- cells using whole blood CD45 kit, and analyzed by standard qRT-PCR. Expression of PLEK2 and C1QB was normalized to the housekeeping gene GAPDH. Data represent results from 6 healthy individuals. The data are shown as the means ± S.E. (*n* = 6). **, *p*<0.01; ***, *p*<0.001 compared with CD45^−^ subpopulation.

**Figure 4:**
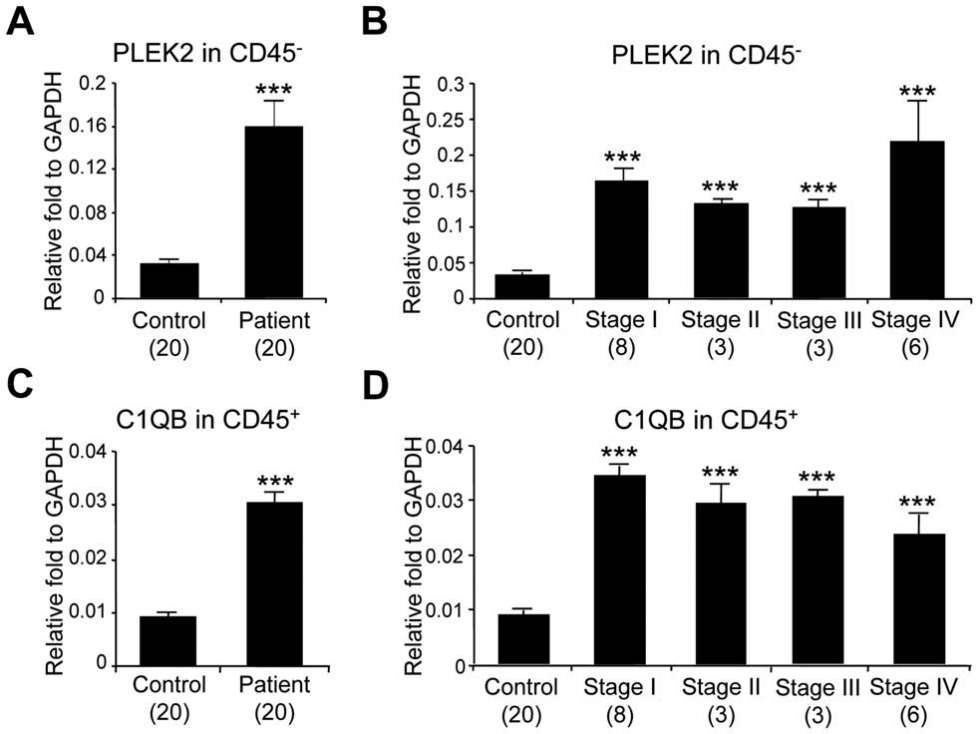
Expression of PLEK2 and C1QB in fractionated blood of melanoma patients and healthy control individuals. Freshly collected blood cells were fractionated into CD45^+^ and CD45^−^ cells using whole blood CD45 kit, and analyzed by standard qRT-PCR. The expression of signature gene PLEK2 in CD45^−^ subsets was compared between healthy individuals (*n* = 20) and melanoma patients (*n* = 20) (**A**) and melanoma from stage I (*n* = 8), stage II (*n* = 3), stage III (*n* = 3) and stage IV (*n* = 6) (**B**). The expression of signature gene C1QB in CD45^+^ subsets was compared between healthy individuals (*n* = 20) and melanoma patients (*n* = 20) (**C**) and melanoma from stage I (*n* = 8), stage II (*n* = 3), stage III (*n* = 3) and stage IV (*n* = 6) (**D**). The data are shown as the means ± S.E. ***, *p*<0.001 compared with control.

The genes (PLEK2 and C1QB) identified in this study have not been well investigated in cancer biology. Due to the nature of biomarker discoveries using high-throughput methodologies and whole blood analysis, the physiological relevance and biological significance of these genes are unclear. Therefore, in order to understand the molecular mechanisms of gene regulation of PLEK2 and C1QB, we analyzed cell subsets that are responsible for the expression of these genes in the blood. Whole blood from 6 healthy control subjects was fractionated into leukocytes (white blood cells) and non-leukocytes (erythrocytes, platelets and other circulating cells) using human CD45 whole blood microbeads. RNA from CD45^+^ and CD45^−^ cell subsets from healthy control individuals were analyzed for the expression of PLEK2 and C1QB by standard qRT-PCR at the University of Colorado Denver. PLEK2 was found to be almost exclusively expressed in CD45^−^ blood cells (Fig. 3, left panel). On the contrary, C1QB showed higher expression levels in CD45^+^ cells compared with CD45^−^ cells (Fig. 3, right panel). We then compared expression of PLEK2 in CD45^−^ cells and that of CIQB in CD45^+^ cells using blood samples from 20 melanoma patients and 20 healthy control individuals by standard qRT-PCR analysis (Fig. 4). PLEK2 expression in CD45^−^ cells was over 3-fold higher in melanoma patients compared with healthy individuals (Fig. 4A). Although sample numbers from each stage were small (n = 3 to 8), upregulation of PLEK2 expression in CD45^−^ subset was detected in blood samples of all stages of melanoma patients (Fig. 4B). On the other hand, C1QB was upregulated in CD45^+^ subset in melanoma patients from all stages compared with healthy individuals (Fig. 4C and 4D).

## Discussion

Practical profiling methodology for biomarkers must be simple, robust, reproducible, and easily accessible with adequate sensitivity and specificity. There is a growing demand for the development of new biomarkers using specimens that are easily and sequentially available, i.e. peripheral blood, urine, or saliva. Peripheral whole blood is a “nucleic acid-rich” and “inflammatory cell-rich” information reservoir, and represents many systemic processes including immune responses and cellular communications. By using RNA stabilization tubes and the globin reduction technology, we identified unique genes by microarray. By using high-throughput qRT-PCR and STEP analysis, we identified unique signature models differentially expressed in the blood of melanoma patients.

The single most discriminating gene was PLEK2 (*p*-value 0.00079 from microarray analysis and 4.2E-22 from high-throughput qRT-PCR). This gene is highly upregulated in the blood of melanoma patients from all stages, and even as a single gene it classifies melanoma and control individuals accurately. Furthermore, studies using fractionated blood cells revealed that PLEK2 was almost exclusively expressed in CD45^−^ cells, indicating the importance of analyzing whole blood cells for profiling blood transcriptomes. PLEK2 is a widely expressed paralog of pleckstrin and was cloned in 1999 [49]. PLEK2 is associated with cytoskeletal rearrangement and cell spreading, and its overexpression results in the production of large lamellipodia, cytoskeletal actin projections on the mobile edge of the cell [49], [50]. Lamellipodia are involved in angiogenesis in endothelial cells and metastasis in melanoma cells [51], [52], [53], [54]. It is thus tempting to consider that increased expression of PLEK2 in blood cells of melanoma patients may provide an environment for tumor growth and metastasis by inducing the production of lamellipodia in circulating cells.

C1QB, the second major classifier gene identified in our study, is a component of the classical complement pathway and is involved in the immune response after injury [55]. Deficiency of C1q has been associated with autoimmune diseases such as lupus erythematosus and glomerulonephritis [56], and upregulation of C1QB was reported in infiltrating immune cells of stomach and breast carcinoma [57] and in the brain of COX-2 transgenic mice [58]. However, the function of C1QB in tumor biology and tumor immunology is still largely unknown. In the current study, we found that C1QB expression was significantly increased in the leukocytes of melanoma patients from all stages. Furthermore, we found that melanoma conditioned medium induced upregulation of C1QB in CD45^+^ cells (data not shown). The findings are in agreement with our hypothesis that peripheral whole blood from cancer patients may represent immune responses and cellular communications altered by the presence of melanoma cells. Further analysis of C1QB, PLEK2 and the other validated biomarkers in fractionated blood cells will provide us more insight into their functions and roles in melanoma development and progression.

Single gene models had been traditionally used as biomarkers. However, advances in high-throughput technologies, development in biostatistics, bioinformatics and data visualization have enabled the generation of molecular patterns (‘fingerprints’, ‘signatures’ or ‘classifiers’), which can be more robust and accurate as biomarkers than single-molecule markers. Unlike single gene models, multi-gene sets are less likely to be influenced by the variation in the expression of one gene, since they use the entire set of genes to classify samples. In the current study, we identified eighteen 2-gene models to correctly classify melanoma patients from healthy control individuals using STEP analysis. Of these, the PLEK2-C1QB signature led to the best discrimination between melanoma and healthy individuals. Noticeably, 2-gene signature models do not always consist of two genes with the lowest *p*-value. For example, PLEK2 was the gene with the lowest *p*-value but C1QB did not have the second lowest *p*-value. In fact, NEDD4L, the gene with the second lowest *p*-value, failed to be selected in the top 30 genes when PLEK2 was used as a starting gene and the combination of PLEK2 and NEDD4L was not included in eighteen 2-gene signatures. It is possible that PLEK2 and C1QB may belong to independent pathways; therefore, changes in expression of both genes may increase reliability in detecting melanoma patients. Likewise, PLEK2 and NEDD4L may function in closely related pathways, making this combination not stronger compared with a single PLEK2 model. Indeed, we showed that PLEK2 and C1QB represent processes in CD45^−^ and CD45^+^ subsets, respectively. These results indicate that signature genes from different pathways or compartments would be more efficient in distinguishing melanoma patients from healthy control individuals.

Our microarray analysis also identified IL-4, Wnt, and Notch signaling pathway in the blood from melanoma patients compared with healthy control individuals. IL-4 is the key cytokine in humoral and adaptive immunity. IL-4 has been shown to be significantly upregulated in tumor infiltrating lymphocytes of cervical carcinoma patient [59]. Th2 response, resulting in IL-4 production, was reported to be enhanced in the peripheral blood of patients with bladder and colorectal cancer compared with healthy controls [60]. Thus, our results showing IL-4 signaling alternation in melanoma patients is consistent with the notion that Th2 immune responses are associated with tumor promotion and progression [61]. On the contrary, Wnt and Notch signaling pathways are activated and expressed in cancer cells, and are well known to play important roles in the development and progression of cancers [62], [63]. These results suggest that melanoma development and survival is associated with changes both in host stromal cells and tumor cells in the local tumor microenvironment and systemic circulation in the blood. Furthermore, gene ontology analysis revealed diverse responses (responses to stimulus, stress and organism) and immune processes (immune process and response, chemotaxis and MHC protein complex), supporting our hypothesis that systemic processes including immune responses and cellular communications can be detected in the blood of cancer patients.

Developing analytical methodologies to detect molecular biomarkers in peripheral blood, which is easily accessible and noninvasive, is highly valuable for the diagnosis and risk management of cancer patients. The current study provides the first analysis of whole blood-based molecular biomarkers for malignant melanoma. Our findings support the hypothesis that gene expression profiles can define subsets of individuals with melanoma. Furthermore, genes and signatures identified in the current study are unique and have not been identified in other studies of blood-based molecular biomarkers. In particular, PLEK2, the strongest gene to classify melanoma patients, was expressed in CD45^−^ subsets, illustrating the importance of analyzing whole blood cells for biomarker studies. It is not clear from these initial studies what mechanisms are involved in the observed changes in gene expression. The fact that the observed differences were with all stages of melanoma suggests that these changes in peripheral blood cells can, and do occur with small tumor burdens. This suggests that such techniques could be used for both early detection of melanoma and monitoring of patients undergoing treatment for residual disease. Whole blood-based transcriptome profiles may also be used to support staging patients, predicting prognosis and predicting response to therapy in the future.

## Supporting Information

Figure S1 **Pathway analysis predicted IL-4 as one of the differentially expressed signaling pathways between normal controls and melanomas.** Significant pathways (*p*<0.05) were analyzed using differentially expressed transcripts (*p*<0.05, fold >1.8). Analysis was performed using pathway analysis tool from GeneSpringGX 10.0. The figure represents IL-4 signaling pathway where 20 differentially expressed genes were highlighted with blue circles.
(TIF)

Figure S2 **Identification of differentially expressed genes from 4 melanoma patients (stage IV, with no treatment, M1–M4) and 6 healthy control individuals (C1–C6).** **A.** Heatmap of 63 selected transcripts with 1.8-fold or greater significant differences between healthy controls and melanomas and a high score by comparison replicates. Colored spots indicate significant up- (red) or down- (blue) regulated genes. Sample tree originated from the clustering of values with Euclidean distance analyzed by GeneSpringGX 10.0. **B.** Transcripts showing 5.5-fold or greater differences between healthy controls and melanomas. Each color represents a single gene.
(TIF)

Table S1 **Differentially expressed gene identified by microarray analysis.**
(DOC)
