# Supplementary figures and images for: Transcriptome Profiling of Whole Blood Cells Identifies PLEK2 and C1QB in Human Melanoma
Source: PLoS One. 2011 Jun 15;6(6):e20971. doi: 10.1371/journal.pone.0020971 (PMC3115966; doi:10.1371/journal.pone.0020971)

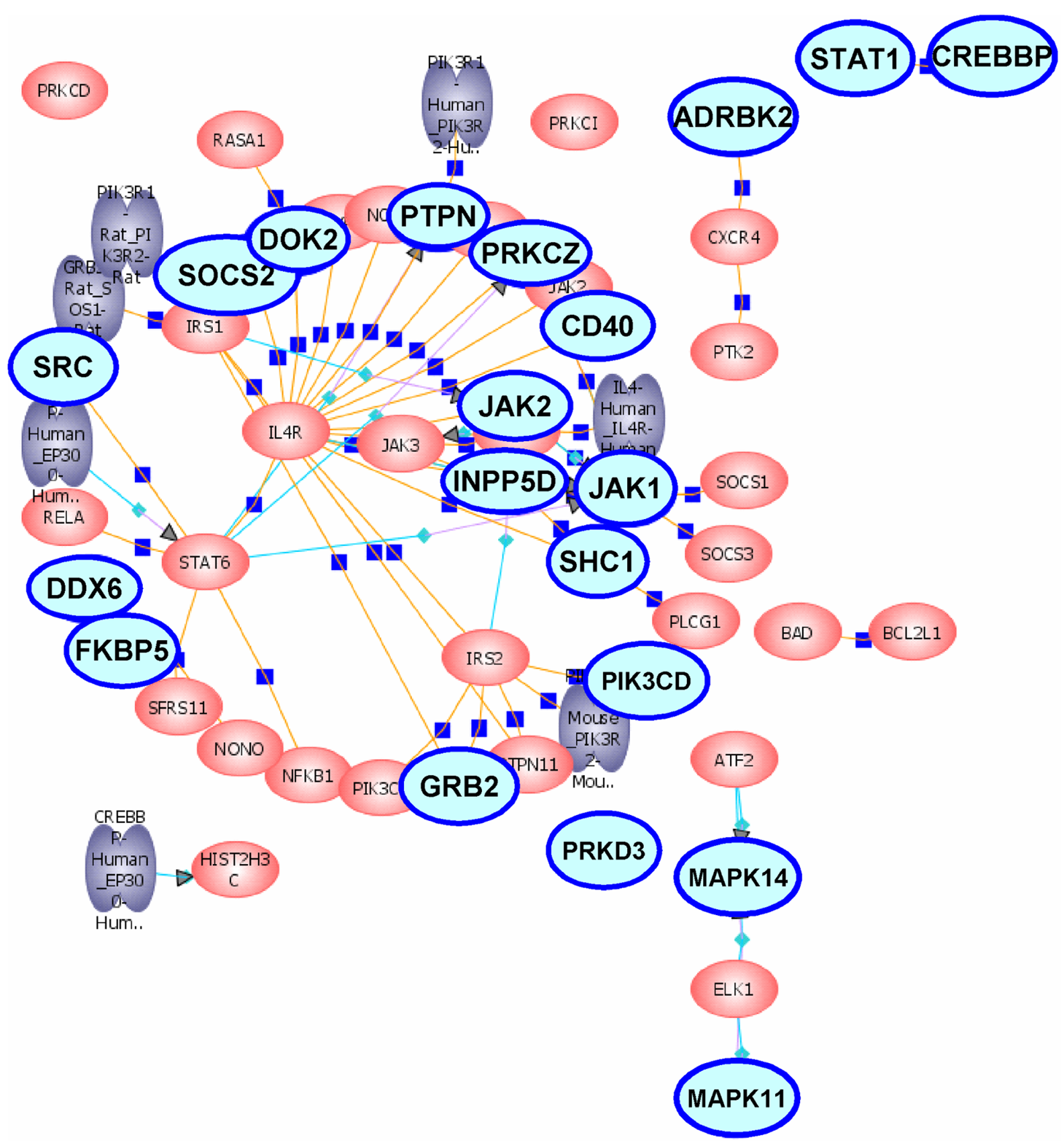

Supplement: Figure S1 — Pathway analysis predicted IL-4 as one of the differentially expressed signaling pathways between normal controls and melanomas. Significant pathways (p<0.05) were analyzed using differentially expressed transcripts (p<0.05, fold >1.8). Analysis was performed using pathway analysis tool from GeneSpringGX 10.0. The figure represents IL-4 signaling pathway where 20 differentially expressed genes were highlighted with blue circles. (TIF) [file pone.0020971.s001.tif]

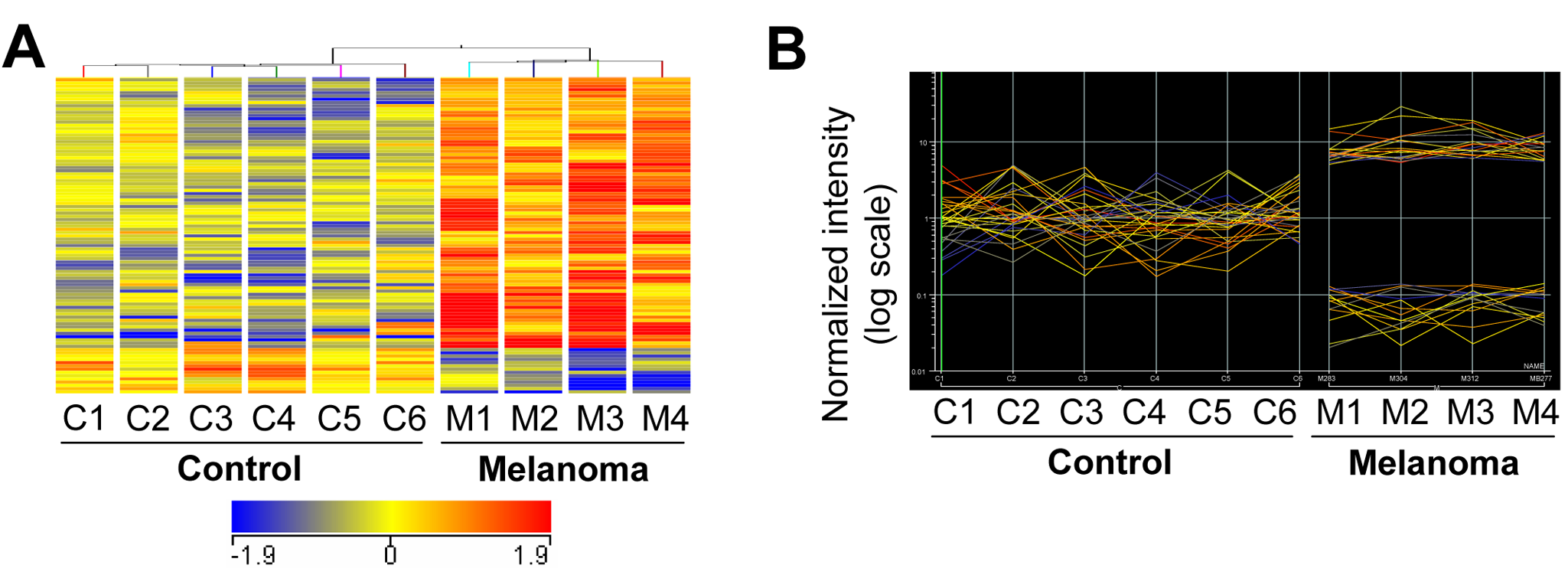

Supplement: Figure S2 — Identification of differentially expressed genes from 4 melanoma patients (stage IV, with no treatment, M1–M4) and 6 healthy control individuals (C1–C6). A. Heatmap of 63 selected transcripts with 1.8-fold or greater significant differences between healthy controls and melanomas and a high score by comparison replicates. Colored spots indicate significant up- (red) or down- (blue) regulated genes. Sample tree originated from the clustering of values with Euclidean distance analyzed by GeneSpringGX 10.0. B. Transcripts showing 5.5-fold or greater differences between healthy controls and melanomas. Each color represents a single gene. (TIF) [file pone.0020971.s002.tif]
