# Supplementary material for: Transcriptome Profiling of Whole Blood Cells Identifies PLEK2 and C1QB in Human Melanoma
Source: PLoS One. 2011 Jun 15;6(6):e20971. doi: 10.1371/journal.pone.0020971 (PMC3115966; doi:10.1371/journal.pone.0020971)
Supplement: Table S1 — Differentially expressed gene identified by microarray analysis. (DOC) [file pone.0020971.s003.doc]

**Table S1. Differentially expressed gene identified by microarray analysis**

| **Category** | **Gene** |
| --- | --- |
| Upregulated genes | ABCC13, ACOX1, AFF1, BLVRB, BPGM, C1QB, C20orf108, CARD12, CCND2, CHPT1, CXCL16,F5, GLRX5, GYPA, GYPB, IGF2BP2, IL13RA1, IL1F7, IL1R2, IQGAP1, IRAK3, LGALS3, N4BP1, NEDD4L, NEDD9, NOTCH2, NPTN, NUDT4, PBX1, PGD, PLAUR, PLEK2, PLXDC2, RAB2B, RAP2C, RBMS1, SIAH2, SLA, SLC4A1, SMCHD1, SNCA, TMOD1, TNS1, TSPAN5, WFDC6, XK, ZDHHC2 |
| Downregulated genes | BCNP1, C18orf24, CDC23, CELSR1, CNKSR2, COBLL1, CXXC6, CYP8B1, EDIL3, HECTD2, INPP4B, KCNK2, LARGE, MEOX2, MGAT5B, MTA1, MTUS1, NBEA, NUCKS1, PAWR, PLEKHQ1, PRRT1, PTPRK, RASGRP3, SCAND2, SCN3A, ST6GALNAC5, TIMELESS, TLK2, ZBTB10, ZC3H7B |
